# Systematic review: characteristics of myocarditis followed by fixed drug eruption and dry eye syndrome in patients who have been vaccinated with monkeypox in children and adults

**DOI:** 10.3389/fcvm.2024.1505298

**Published:** 2025-01-29

**Authors:** R. Mohamad Javier, Stephanie Angela Prijanto, Vallexa Septina Yora, Desak Gede Yuliana Eka Pratiwi, Errini Sabilla Lilhawa Ditsi, Bella Adelia, Verika Christabela Tansuri, Hendry Wijaya, Georaldhy Yussufy Caecarma, Intan Trikumala Damayanti, Anisa Ramadhanti, Atika Rahmaputri, Chabib Fachry Albab, Afif Ferdian, Fatih Farabi, Fadila Risang Ayu, Ni Putu Ika Regina Maharani, Andika Prasetyo Arifin, Eko Setyo Herwanto, Saidah Rahmat A, Safira Dita Arviana, Syifa Nur Lathifah, Nanda Rizki Yulinar, Laksmitha Saktiono Safitri, Basyar Adnani, M. Rizki Fazrian Danu, Natasya Naomi, Dayu Dwi Deria, Aulia Syifa, Panca Andana, Adrian Prasetya, Fachira Rachel Agfata, Magistra Cylvia Margaretha, Subandono Bambang Indrasto, Sukirman, Rachmanto, Hayatun Nufus, Pertiwi Febriana Chandrawati, Aan Dwi Prasetyo, Lucky Sutanto, Moch Aleq Sander

**Affiliations:** ^1^ Department of Internal Medicine, Gatot Soebroto Army Hospital, Jakarta, Indonesia; ^2^ Faculty of Medicine, Widya Mandala Surabaya Catholic University, Surabaya, Indonesia; ^3^ Faculty of Medicine, Muhammadiyah Malang University, Malang, Indonesia; ^4^ Faculty of Medicine, Brawijaya Univeristy, Malang, Indonesia; ^5^ Faculty of Medicine, Pelita Harapan University, Tangerang, Indonesia; ^6^ Faculty of Medicine, Atma Jaya Indonesian Catholic University, Jakarta, Indonesia; ^7^ Faculty of Medicine, Airlangga University, Surabaya, Indonesia; ^8^ Department of Internal Medicine, Permata Bunda Hospital, Malang, Indonesia; ^9^ Department of Internal Medicine, Heartology Cardiovascular Hospital, Jakarta, Indonesia; ^10^ Faculty of Medicine, Diponegoro University, Semarang, Indonesia; ^11^ Faculty of Medicine, Hang Tuah Surabaya University, Surabaya, Indonesia; ^12^ Faculty of Medicine, Krida Wacana Christian University, Jakarta, Indonesia; ^13^ Faculty of Medicine, Maulana Malik Ibrahim Malang University, Malang, Indonesia; ^14^ Department of Internal Medicine, Bayu Asih Purwakarta Hospital, Purwakarta, Indonesia; ^15^ Faculty of Medicine, Jember University, Jember, Indonesia; ^16^ Faculty of Medicine, Malikussaleh University, Aceh, Indonesia; ^17^ Faculty of Medicine, Islamic University of Indonesia, Yogyakarta, Indonesia; ^18^ Department of Opthalmology, Gatot Soebroto Army Hospital, Jakarta, Indonesia; ^19^ Department of Pediatric, Gatot Soebroto Army Hospital, Jakarta, Indonesia; ^20^ Department of Internal Medicine, Persahabatan Hospital, Jakarta, Indonesia; ^21^ Department of Pediatric, Muhammadiyah Malang University Hospital, Malang, Indonesia; ^22^ Department of Neurology, Muhammadiyah Malang University Hospital, Malang, Indonesia; ^23^ Department of Obstetrics and Gynecology, Kartika Husada Tanjungpura, Tanjungpura, Indonesia; ^24^ Department of Surgery, Muhammadiyah Malang University Hospital, Malang, Indonesia

**Keywords:** monkeypox, myocarditis, dry eye syndrome, fixed drug eruption, MPOX vaccination, characteristics, diagnosis

## Abstract

**Background:**

The Monkeypox Virus (MPOX) has caused a surge in viral infections, leading to the WHO recognizing it as a public health emergency of international concern. MPOX infection shares clinical similarities with smallpox but can cause complications like myocarditis, anorectal pain, ocular lesions, kidney damage, or soft tissue superinfection. The study aims to understand the characteristics of myocarditis, fixed drug eruption, and dry eye syndrome in Monkeypox patients.

**Methods:**

This review was conducted based on PRISMA (Preferred Reporting Items for Systematic Reviews and Meta-Analyses), and the Cochrane Handbook for Systematic Reviews of Interventions. The data was obtained from Scopus and international journal databases by conducting combined keyword searches restricted to English-language publications.

**Result:**

The study examined 25 cases of Monkeypox, primarily involving males aged 32.9 years and experiencing chest pain. The prognosis was generally good, with no reported death. Risk factors for infection include sexual activity, STD diagnosis, sexual encounters, and workplace exposure to orthopoxviruses. Most cases were male and involved chest pain. Myocarditis, an inflammation in the myocardium, can cause dilated cardiomyopathy, acute arrhythmia, and heart failure. The pathophysiology of myocarditis in Monkeypox patients is not yet determined due to rarity of cases.

**Conclusion:**

MPOX infection presents unique complications like myocarditis, necessitating research for vaccines, antiviral drugs, and infection prevention measures. Early screening for chest pain and investigating MPXV infection's pathogenesis and clinical features are crucial for differential diagnosis during outbreaks. This systematic review can determine MPOX infection outcomes and prepare appropriate treatment for patients with complications.

## 1 Introduction

There has been an increase in the incidence of malignant viral infections caused by the monkeypox virus (MPOX) since the end of the global COVID-19 pandemic. From outbreaks occurring sporadically in Africa to affecting around two-thirds of the world's countries, On July 23, 2022, the MPOX outbreak was declared by the World Health Organization (WHO) as a public health emergency of international concern (PHEIC). In 1970, the first cases of MPOX were reported in children in the Democratic Republic of Congo (Zaire), after being previously reported in monkeys in 1959 (1). On January 5, 2023, there were 84,318 confirmed cases of MPOX in 110 countries, with 103 countries unreported. The highest cases were reported in the United States (*n* = 29,913), followed by Brazil (*n* = 10,544) and Spain (*n* = 7,500). Previously, in Africa, MPOX infection outbreaks were reported sporadically after contact with wild rodents. Despite large local outbreaks and transmission associated with travel outside of Africa, secondary spread has been limited, indicating limited human-to-human transmission. The recent MPOX outbreak has opened the floodgates for extensive research to determine the reasons behind limited transmission, even in highly endemic areas where the viral blood load is high (2).

MPOX virus infection clinically resembles smallpox infection with an incubation period of usually 6–13 days but can range from 5 to 21 days. In addition to systemic signs and symptoms, MPOX infection causes a variety of ophthalmic manifestations. Although MPOX is a self-curable disease, it can cause permanent visual sequelae (3). Systemic MPOX shows up as feverish, papular, pustular, vesiculopustular, and ulcerative sores on the face and trunk, along with swollen lymph nodes. The current epidemic primarily affects men who have sex with multiple partners. Clinical features include skin lesions, systemic signs, and less commonly, skin superinfection, or severe and painful anorectal or ophthalmic involvement (4). Even though MPOX infections sometimes go away on their own, some patients need to be admitted to the hospital because of complications such as myocarditis, severe anorectal pain, eye lesions, kidney damage, or soft tissue superinfection. MPOX may increase the risk of immune-mediated heart damage and myocarditis in this organ (5).

Myocarditis is a focal or diffuse inflammation of the myocardium that can cause acute arrhythmias, dilated cardiomyopathy, and heart failure. Viruses such as adenovirus, parvovirus B19, HIV, or enterovirus are known to cause myocarditis. Monkeypox has only been responsible for five cases of myocarditis, all of which occurred in 2022 during the current epidemic and had various possible causes (6).

Elevations in cardiac biomarkers associated with chest pain symptoms were observed in case reports of myocarditis due to monkeypox. Lymphocytic inflammation followed by myonecrosis is the most common pathophysiology of viral myocarditis, although the specific pathophysiology of smallpox virus myocarditis remains an area of research. Monkeypox-associated viral pericarditis presents with mild pericardial effusion and is recommended to be suspected in patients who demonstrate electrocardiographic changes in ST elevation associated with chest pain. The published case report is of a patient who was not vaccinated against the orthopox virus and was otherwise healthy with no immunocompromised conditions. Interestingly, myopericarditis also occurs as an adverse effect of the smallpox vaccination (7).

Typically, MPOX prognosis is good, and treatment is usually symptomatic, with only 4% of patients requiring antiviral drugs. However, some patients may experience severe infections that affect organs such as the eyes. It is true that this disease has the potential to cause dry eye disease and even blindness in one or both eyes, especially in the pediatric population. Therefore, it is important to characterize the ophthalmic manifestations of MPOX virus infection, as these are associated with more severe disease symptoms, thus being an indication for antiviral therapy and hospitalization (8).

Cardiac involvement in dry eye syndrome generally consists of an acute form of myocarditis: hypersensitivity myocarditis or acute necrotizing eosinophilic myocarditis (ANEM). Although a variety of medications, such as anticonvulsants and sulfonamides, have been associated with dry eye syndrome, there is little data available regarding the association between dry eye disorder-associated myocarditis and medications used to treat dry eye disorders (9).

Severe drug adverse reactions can occur and be characterized by fever, erythematous eruption, eosinophilia, atypical lymphocytosis, lymphadenopathy, and dysfunction of multiple organs, including the heart. Drug erruption is inflammatory reaction of the skin and mucosa caused by the ingestion of a drug into the human body via one of the routes of administration (10). Severe cutaneous adverse drug reactions (SCAR) not only appear suddenly and cause extensive and serious skin lesions, which can even affect the oral mucosa, but can also cause symptoms of systemic poisoning involving multiple organs, serious damage to liver and kidney. Function, and even life-threatening conditions, such as severe drug stimulation (11).

Severe drug eruption may cause cardiac involvement, such as myocarditis, and cause fatal damage. While etiology of severe drug eruptions in MPOX mayhap related with smallpox vaccine, it still remains unclear. Thus this review aim to analyze the latest literature and cases assessing myocarditis followed by drug eruption and dry eye syndrome in vaccinated-MPOX patients.

## 2 Literature review

### 2.1 Monkeypox general manifestation

While MPXV infection shares many clinical characteristics with smallpox, it also presents with unique symptoms such lymphadenitis, which is particularly common in the cervical, submandibular, and inguinal regions. Typically, MPXV infections take five to twenty-one days to manifest symptoms. The illness itself has a natural healing process, but in certain situations-such as pregnancy and immunocompromised conditions-it can lead to catastrophic illness and even death. Age, gender, and immunization status were revealed to be significant determinants of the severity of MPXV clinical symptoms. Even while the majority of MPOX cases in the present outbreak are minor, some people may develop severe illness or have uncommon or unexpected clinical signs. Fever and skin sores are the most typical symptoms. Nonetheless, a small percentage of patients may develop serious symptoms that include problems in several bodily systems and could even be lethal. Prodromal symptoms include fever, vomiting, malaise, conjunctivitis, and lymphadenopathy. However, there have been reports of several potentially fatal outcomes, including encephalitis, bronchopneumonia, acute kidney injury, proctitis, myocarditis, and sight-threatening keratitis (2). MPXV infections are divided into two main phases:

(1) prodromal phase, lasting 0–3 days and beginning 4–17 days after viral exposure, during which time symptoms include enlarged lymph nodes, fever, headache, chills, exhaustion, back pain, and muscular aches; And
(2) The rash phase, which lasts for 7–21 days and starts three days after prodromal signs (3).

MPOX typically presents with a variety of lesions, such as a maculopapular rash that is itchy or painful and develops into vesiculopustular lesions. The rash is well-defined, around the same size, and it may spread, though it is typically more concentrated on the face and extremities. Additionally, impacted are the genitalia (30%), conjunctiva, oral mucosa, and extremities such the palms and soles of the feet. A new layer of skin or mucosa will emerge beneath the lesion, which often advances from the macular stage in 10 days or fewer to papules, vesicles, pustules, crusts, and scabs before coming off. Furthermore, studies indicate that MPXV infection also results in neurological symptoms, which can range from general ones like headaches and myalgia to more uncommon ones like seizures and encephalitis (1).

Humans might acquire a disease from other humans or from animals. The natural host is in contact or ingested during the initial transmission. The latter happens through skin lesions of infected patients, contact with body fluids, and respiratory droplets. According to a study, 95% of infected cases reported having close sexual contact, and 98% of infected individuals were men who identify as homosexual or bisexual (6). The most common anatomical location of the lesion is the anogenital area, which further supports the idea that sexual contact is the predominant mechanism of transmission. On the other hand, sexual contact transmission has not been verified as a mode of transmission and is presently under investigation. Children and adults with acquired immunodeficiency syndrome (AIDS), impaired health, and those on antiretroviral therapy (ART) are particularly susceptible to transmission and symptoms (12).

### 2.2 Myocarditis

For a long time, smallpox, a virus related to MPXV but far more deadly, was believed to be the cause of myocarditis. Furthermore, since the 1950s, European researchers have documented the cardiac side effects of smallpox vaccination, including post-vaccination myocarditis and myopericarditis. Given their intimate relationship, it becomes sense to believe that MPXV could also have a tropism for cardiac tissue or harm the heart through immunological mechanisms (13). Following smallpox vaccination, myocarditis has also been recorded. In children and teenagers, the virus replicates during vaccine-based vaccination; as a result, cardiac involvement in orthopoxvirus infections has been documented. A number of case reports following COVID-19 have disclosed specific symptoms suggesting a clinical suspicion of myocarditis. Nonetheless, there are multiple cases of myocarditis that have been histologically established, as well as cases of suspected direct viral myocarditis caused by SARS-CoV-2 based on epidemiological circumstances (14).

Myocarditis has a varied histologic shape and variable clinical symptoms, making diagnosis challenging. Clinically suspected myocarditis can be diagnosed with noninvasive tests such as echocardiography, ECG, and standard cardiac magnetic resonance (CMR) abnormalities. Myocarditis is linked to signs and symptoms of chronic or recurring heart muscle injury. Both chronic inflammatory cardiomyopathy and acute myocarditis can result in elevated markers of heart muscle damage as well as persistent or recurrent symptoms. Severe myocarditis can result in high-grade atrioventricular block, ventricular arrhythmias, cardiogenic shock, and other consequences. Myocarditis prognosis is mostly determined by the disease's etiology and stage. Acute myocarditis can either go away on its own in a few weeks or get worse and develop chronic inflammatory cardiomyopathy and persistent heart dysfunction. Furthermore, the illness could worsen and result in fulminant myocarditis, which would require circulatory care and hemodynamic compromise (15).

### 2.3 Pathogenesis

Myocarditis is inflammation of the myocardium that can cause dilated cardiomyopathy, acute arrhythmias, and heart failure. Depending on the type of myocarditis, different treatment outcomes may be necessary. For example, acute myocarditis typically resolves in a few weeks, but chronic inflammatory cardiomyopathy requires long-term care. Fulminant myocarditis, on the other hand, requires circulatory support measures due to severe hemodynamic compromise. Viruses like adenovirus, parvovirus B19, HIV, or enterovirus are known to cause myocarditis. Since there was no indication of a direct viral infection of cardiac cells in the histopathological analyses of samples from recipients of the viral vaccine who developed myocarditis, several researchers have hypothesized that this disease is actually an autoimmune reaction (16).

The primary cause of viral myocarditis, which typically manifests 10–14 days after infection and can either resolve on its own or develop to fulminant myocarditis, is lymphocytic myocarditis with myonecrosis. Heart failure, dilated cardiomyopathy, and cardiac fibrosis are hallmarks of the chronic stage of noninfectious viral myocarditis. Because human occurrences of MPOX-associated myocarditis are rare, the pathogenesis of MPXV-induced myocarditis is still unknown. The majority of myocarditis symptoms are mild and go away on their own. Serious side effects, such dilated cardiomyopathy, are uncommon. One theory for how myocarditis develops in MPOX patients is because MPXV infection of cardiac muscle cells. This virus can infect a variety of host cells, including cardiac myocytes, which can lead to tissue damage and inflammation in the heart, ultimately resulting in cell death (17).

Another explanation for the emergence of myocarditis in MPOX patients could be an immune reaction to the virus. Myocarditis may result from the immune system's production of cytokines and other inflammatory mediators upon identification of a viral infection. In extreme situations, an unchecked immune response sets off a cytokine storm that causes widespread tissue damage, including damage to the heart muscle. In conclusion, it is thought that direct viral infection of cardiac muscle cells and an immunological response to the virus cause myocarditis in MPOX patients. Thus, more investigation is required to fully comprehend the mechanisms underlying the development of myocarditis in MPOX patients (9).

### 2.4 Dermatological manifestation

Skin rash is the most frequent clinical manifestation, occurring in 95% of cases, according to reports. As previously indicated, the anogenital region is the most frequently affected area, followed by the trunk and extremities (65%) and the face (25%). Although vesiculopustular lesions predominate with centrifugal distribution, the rash can be pleomorphic. The lesion is between 0.5 and 1 cm in size, and it normally crusts over in two to three weeks. A patient is deemed non-infectious when they reach the desquamation phase, which is shown by the scab peeling off of them. Proctitis, tonsillitis, pharyngitis, lymphadenopathy, tenesmus, and diarrhoea are other symptoms. In the second week, digestive symptoms appear and cause dehydration. These symptoms are typically made worse by oropharyngeal ulcers because they make it harder to maintain nutrition. Fever, fatigue, myalgia, and headache are examples of nonspecific systemic symptoms that are frequently experienced in the prodromal phase, which begins three days prior to the beginning of the skin rash. Acquired Immunodeficiency Virus (AIDS) co-infection is prevalent, accounting for 41% of cases. The high frequency of homosexual and bisexual behavior could be the cause of this disease (18).

### 2.5 Ocular manifestation (dry eye syndrome)

Ocular symptoms include pain, redness, wetness, photophobia, discharge, swelling around the eyes, and decreased vision in addition to systemic symptoms. The majority of problems, including those related to vision, appeared to affect unvaccinated people more often (74%) than vaccinated people (39.5%). There have been reports of MPOX affecting the sclera, cornea, conjunctiva, and eyelids. MPOX-associated eye disease (MPOXROD) is the umbrella name for the range of ocular symptoms that can be brought on by MPOX infection (19).

There have been several reports of MPOX phenotypes linked to conjunctivitis. There have been reports of vesicular, infiltrative, and ulcerative lesions. Similar to smallpox, ocular ulcers may result from the close-together dissemination of these pustules. Large, uniformly shaped lesions on the conjunctiva can have a pale appearance. Conjunctival thickening and a serpiginous, white appearance characterize infiltrative lesions. There may be mucoid discharge and follicular responses. In patients with MPOX, conjunctivitis may be a sign of more severe systemic illness, as 47% of patients are bedridden vs. 16% in individuals without conjunctivitis. The primary worry is still the possibility of bacterial superinfection exacerbating corneal ulcers. In the end, the latter might cause thinning, anterior staphyloma, or perforation. There's evidence linking this procedure to smallpox. Vitamin supplements and excessive lubricant use are two ways to avoid this (20).

MPOX might take anywhere from 5 to 21 days to incubate. Fever, chills, headache, lethargy, asthenia, swollen lymph nodes, back pain, and myalgia are some of the early warning signs and symptoms. Within one to five days after the fever starts, rashes of different sizes start to form on the face and spread to the hands, legs, and feet. These lesions range in size from 0.5 to 1 cm. The rash develops into macules, papules, vesicles, and pustules before gradually clearing up into crusts and scabs that fade away as the patient heals. The primary characteristic that sets MPOX apart is lymphadenopathy, which can affect the neck, groin, and submandibular region. After direct human-to-human transmission, more severe sickness is linked to high viremia, severe disease, and death. Accompanying infections, respiratory issues, encephalitis, blinding keratitis, and gastrointestinal symptoms like vomiting and diarrhea are among the complications of this illness (21).

## 3 Methods

This review was conducted based on PRISMA (Preferred Reporting Items for Systematic Reviews and Meta-Analyses), and the Cochrane Handbook for Systematic Reviews of Interventions. The retrieval of data focuses on (1) the use of posology (dose and time), (2) the type of treatment, (3) the interpretation of the results, and (4) the side effects of the treatment. If, during the research, there was an inability to report data from one or more of these four data sets, it will still be counted (qualifies) as inclusion, but the missing information data will be recognized.

The MEDLINE (Medical Literature Analysis and Retrieval System Online, via PubMed) and Scopus databases were searched until October 2023 for studies evaluating the use of strontium ions as biomaterial composites in the form of scaffolds in regenerating tissue in the health and medical fields. The search strategy was limited to English language publications using combined keywords such as monkeypox, myocarditis, dry eye syndrome, drug eruption, characteristics, diagnosis, and combinations.

Unpublished data were sought by searching databases listing unpublished studies (OpenGray-www.opengery.eu). Manual searches were also carried out based on the reference lists of selected papers. Electronic databases of the following journals, considered important for this review, were manually searched separately, including Sciencedirect, springer, Taylor Francis, Wiley, NCBI, and PubMed. Furthermore, the bibliographic references of the included studies were also searched for potentially relevant studies.

**Figure 1:**
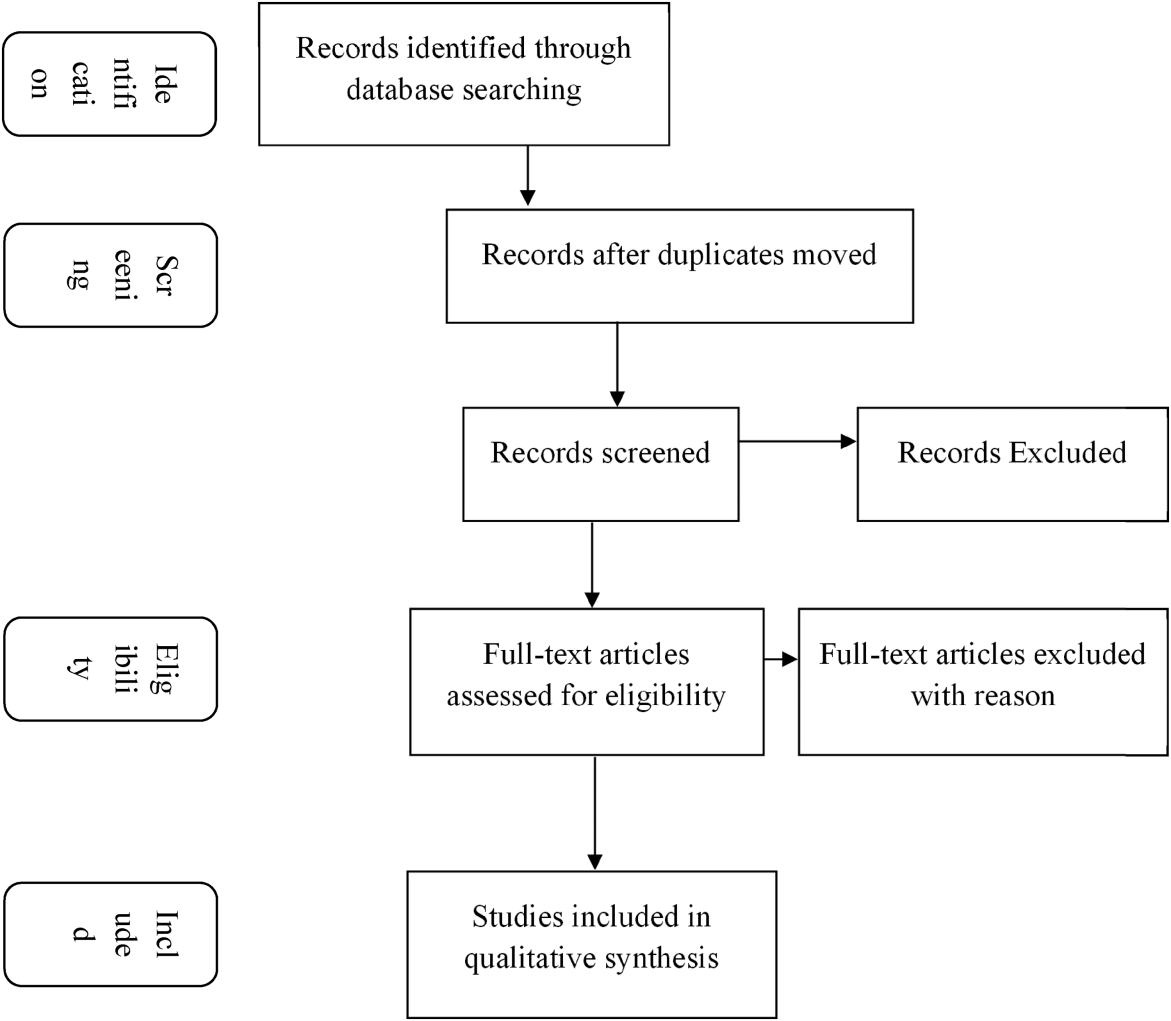
Systematic review flow diagram.

Titles, abstracts, and full texts of search results were screened independently by reviewers. When there were differences of opinion, the reviewers discussed the study and reached a consensus. The PRISMA flowchart delineated the selection processes of all research, as illustrated in Figure 1.

Researchers carried out data extraction and assessed the validity of research that met the inclusion criteria. Data were extracted focusing on the start of treatment, duration of treatment, examination period, evaluation methods and outcomes, according to what was reported in each study (e.g., biocompatibility, cytotoxicity, biomechanics).

Any quantitative and qualitative studies relating to parameters that can explain/describe the characteristics of myocarditis in monkeypox patients with drug eruptions and dry eye syndrome as potential ingredients in diagnosing the tendencies of monkeypox sufferers, determining and how these substances interact with the white blood system are included as a result.

Quality assessment was performed based on the SYRCLE risk of bias tool (no summary score for included studies). Reviewers independently assessed the quality of the studies. Consensus deliberation is carried out if there are differences of opinion regarding the data being assessed.

## 4 Review

### 4.1 Search results

A thorough search from an international journal database and scopus was done and a total 50 studies were identified. After the scanning process there are 31 studies which correlate with the main topic. Based on the abstract and keyword there are 28 studies which potentially could be the criteria for developing the topic we brought in this systematic review. After thorough understanding of the studies, we exclude studies that are not eligible with our inclusion criteria. Considering the exclusion and inclusion criteria, final 25 studies were selected from 50 studies that fulfill our criteria. The following information about myocarditis as an independent variable shows on the table below which includes title of the journal, year publishing, objective of the study, sample and criteria, research instrument, methods or data, and results of studies.

## 5 Discussion

The complete details of the studies included in this systematic review can be seen in Table 1. Twenty-five case studies of monkeypox were examined. Four cases came from the United States, three cases from France, two cases from Canada, and one case each from Portugal, Spain, and Puerto Rico. The patient's average age was 32.9 years, ranging from 21 to 51. All of the patients were male (22). The average duration of symptom onset was 8.4 days, with chest discomfort being the predominant symptom reported by all patients. The patient was treated with numerous medications, including NSAIDs, colchicine, aspirin, nitroglycerin, tecovirimat, and an ACE inhibitor. The evaluation spanned four to thirty days, with an average of 12.5 days. One of the studies lacked assessment information. Every patient exhibits signs of symptomatic recovery (1).

**Table 1:** Data analysis.

No	Journal title and researcher name	Objective	Sample	Research instrument	Methods	results	Journal clustering
1	Acute fulminant myocarditis in a patient with primary Sjögren's syndrome (17)	To describe a patient, woman with pSS who experienced an acute fulminant myocarditis. The Endomyocardial biopsy show source of autoimune	A case study of woman, 35 years old with history of Tiroiditis Hashimoto for 14 years and dry mouth for 6 months	Case study	Endomyocardial biopsy and bleeding management	The conclusion, as long as we know this case study is the first case study that describe the pathology of myocarditis related to pSS. in pSS, the infiltration of T cell may play important rule of pathogenic part in developing myocarditis.	Scopus
2	Clinical features and management of human monkeypox: a retrospective observational study in the UK (22)	To describe the long-term clinical features of *Monkeypox* in high-income population, and in addition of dynamics virus, and all the side effects related to the newantiviral therapy	All of the patients in the centre of High Consequence Infectious Disease (HCID) in Liverpool, London, and Newcastle, which coordinated by national HCID system	Clinical features	Medical reports review	A patient is a healthcare worker that infected through nosocomial and another patient who got viral infection from abroad transmit the infection to adults and children in their household.	Scopus
3	Dry eye syndrome in patients with cardiovascular pathology (38)	To disclose the new perspectives to treat patient with dry eye syndrome by biometric analysis.	In this study involved 200 patients of Comanesti County Hospital.	Blinded-randomized clinical trials	The cases arranged in 4 groups in each group consist of 50 patients, with or without cardiovascular disease, with or without dry eye syndrome.	Heart failure significantly happened more often in women and they who 60 years old or above assume to have 2.5 times higher risk in the entire groups and around 2 times higher in patient who have dry eye syndrome.	Scopus
4	Myocarditis in monkeypox-infected patients: a case series (16)	To specifies case of myocarditis caused by *Monkeypox*, a poorly explained condition	3 cases myocarditis in France, 2022	Case study	The dysfunction in reporting descriptive case series	Patient is a grown man without medical history of skin lesions with positive polymerase chain reaction to *Monkeypox* virus. A few days after sign of skin appear, patient develop acute chest pain, increase of cardiac marker, and inflammatory syndrome that match with myocarditis.	Scopus
5	Treatment of ocular-involving monkeypox virus with topical trifluridine and oral tecovirimat in the 2022 monkeypox virus outbreak (26)	To report ocular involving monkeypox infection cases in USA during the outbreak in 2022	A 36 years old man with HIV infection in controlled came to emergency department	Case study	Case-presentation analysis	Data about clinical features and course of the illness, especially related to ocular manifestation is restricted	Scopus
6	Characteristics and Management of Ocular Involvement in Individuals with Monkeypox Disease (30)	To find the correlation of MPOX polymerase chain reaction quantification and severity of ocular manifestations.	5 individual patients	Systematic review	Case-presentation analysis	By using standardized ophthalmology diagnostic assessment and management based on protocol, we observe ocular involvement rarely happen, however it may be severe in this MPOX outbreaks. In case of severe case with or without ocular involvement, tecovirimat is a treatment that should be considered.	Scopus
7	Refractory cardiac myocarditis associated with drug rash with eosinophilia and systemic symptoms syndrome due to anti-bipolar disorder drugs: a case report. (28)	To find out myocarditis mortality rate as acute necrotizing eosinophilic myocarditis (ANEM).	37 years old man with myocarditis associated with DED syndrome.	Case report	Case presentation	The heart function is temporarily normalized by using high dose of prednisolone. However, the inflammation persist as indicated by the increase of troponin T and decrease of ejection fraction of left ventricular after several months.	Scopus
8	Acute Myocarditis A New Manifestation of Monkeypox Infection?. (15)	To verify acute myocarditis as the new manifestation of monkeypox infection.	A 31 years old male patient with confirmed monkeypox infection	Case report	Case presentation	This case highlighting the cardiac involvement as the potential complication related to monkeypox infection	Scopus
9	Monkeypox – An emerging pandemic (5)	To be noted that patient with weak immune system, pregnant woman, children under 8 years old must be given prophylaxis before and after exposure to the vaccine	A 26 years old polygamous homosexual man with history of syphilis.	Case report	Case presentation	Patient with severe symptom or respiratory complication could be treat with antivirus such tecovirimat (TPOXX) and brincidofovir or intravenous immunoglobulin vaccines (VIGIV)	Scopus
10	Outcomes of radiofrequency catheter ablation for persistent and long-standing persistent atrial fibrillation. (10)	To asses 1 year results of patient with long-standing persistent atrial fibrillation (AF) that has been treated with catheter ablation process.	67 patients involved. (40% with long-standing persistent AF)	Case study	Retrospective observational study	Catheter ablation is an effective treatment for patient with persistent AF and those who undergo special approach showed lower arrhythmia recurrence	Scopus
11	Communicating coronary and ventricular pseudoaneurysms complicating coronary artery perforation. (1)	To asses communicating coronary and ventricular pseudoaneurysms	A 75 years old man experienced recurrent bleeding after surgical treatment	Case report	Case presentation	Interrelated complication of communicating coronary and ventricular pseudoaneurysms. Cutaneous intervention to seal the site of repeated bleeding by implanting covered stent successfully managed this rare pseudoaneurysms.	Scopus
11	A case of monkeypox in a sexually-active man with HIV and syphilis. (4)	To find out that antiviral therapy is available for patients at risk of developing severe disease	19 years old and 27 years old man who sexually active	Case report	Case presentation	Patient begin antiretroviral therapy after being diagnosed with HIV recently. Patient given intramuscular penicillin g benzathine for syphilis treatment, and are given referral for follow up on infectious disease.	Scopus
12	Eczema monkeypoxicum: Report of monkeypox transmission in a patient with atopic dermatitis. (3)	To describe WACM case that related to eczema.	A 63 years old man with DA on his hand since he was little came to dermatology outpatient unit.	Case report	Case presentation	The influence of disseminated monkeypox due to DA superinfection on severity of the disease remain unclear.	Scopus
13	The outbreak of monkeypox 2022. (12)	To report the outbreak of monkeypox	92 cases were reported in those aged 20–50 years.	Case report	Case presentation	There is no definite treatment of MPX. CDC recommends administering the smallpox vaccine in 4 days after exposure can prevent the disease and in 2 weeks can decrease the symptom severity.	Scopus
14	Poxviruses and the immune system: Implications for monkeypox virus. (36)	To develop an outbreak of smallpox-like infections in humans	Monkeypox cases between May 13th, 2022 until June 24th, 2022.	Case report	Case preentation	Many evasion mechanisms are highlighted, that may have implications for designing specific immunotherapies for PXV in the future	Scopus
15	Myocarditis in athletes recovering from COVID-19: a systematic review and meta-analysis	To assess the incidence rate of myocarditis detected by Cardiac Magnetic Resonance (CMR) in athletes recovering from COVID-19	7,988 athletes from 15 studies	Case report	A Systematic literature review	The prevalence of COVID-19-associated myocarditis in the athletic population ranges from 1 to 4%. Although the incidence rate is quite low, current screening protocols are a useful instrument for the athletes return safely for play and to properly manage CMR studies	Scopus
16	Current perspectives on severe drug eruption. (25)	To Understand the clinical characteristics, treatment, and prognosis of severe drug eruptions from sensitizing drugs	2 to 7 million cases per year	Case report	Case presentation	The pathogenesis of severe drug eruptions is still being explored. Early diagnosis and treatment of severe drug eruptions is critical to the patient's prognosis, and attention should be paid to possible long-term sequelae.	Scopus
17	Diagnostic accuracy of cardiovascular magnetic resonance in acute myocarditis. (21)	to explore the diagnostic accuracy of various cardiovascular magnetic resonance (CMR) index tests for the diagnosis of acute myocarditis in adult patients	22 studies	Diagnostic Accuracy Study Quality Assessment Tool-2	All diagnostic cohort studies and case-control studies	The new CMR mapping technique provides high diagnostic accuracy for the diagnosis of acute myocarditis and is a promising successor to the classic elements of LLC for routine diagnostic protocols.	Scopus
18	Early characteristics of fulminant myocarditis vs non-fulminant myocarditis	to identify baseline characteristics of FM compared with non-fulminant myocarditis (NFM).	158 FM patients and 388 NFM patients.	Case report	Case presentation	Lower SBP, higher CK, wider QRS duration, lower LVEF, thicker LVPWd, higher incidence of ST depression, VT/VF and syncope as well as lower incidence of chest pain are early characteristics of FM.	Scopus
19	Monkeypox virus immune evasion and eye manifestation: beyond eyelid implications. (11)	To specify the various ocular manifestations and immune evasion associated with monkeypox virus infection and its complications.	Monkeypox cases between year 2021 and 2022	Case report	Case presentation	Orthopoxviruses can evade the host immune response by secreting proteins that antagonize the function of the host chemokines IFN*γ*, CC and CXC, IL-1β, and the complement system.	Scopus
20	Ophthalmic manifestations of monkeypox infection. (29)	To discuss MPX morphology, various modes of transmission, routes of viral infection, and host immune response	Children and teenager with Monkeypox in between year 2021 and 2022	Case report	Case presentation	This disease can be life-threatening, and special attention needs to be given to children, the elderly, and pregnant women as they are more vulnerable.	Scopus
21	Spectrum of ophthalmic manifestations in monkeypox virus infection worldwide. (24)	To determine which manifestations of ocular disease are associated with more severe cases	Children and teenager with Monkeypox in between year 2021 and 2022	Case study	Ophthalmic manifestation	Ophthalmic manifestations, such as conjunctivitis and periocular umbilical lesions, are the most common in Mpox virus infection.	Scopus
22	Emergence and dissemination of monkeypox, an intimidating global public health problem. (2)	To provide an overview of this neglected zoonotic pathogen.	Two different groups, Central Africa and West Africa, with mortality rates of 10.6% and 3.6% respectively.	Case report	Case presentation	The use of non-pharmaceutical interventions and immunizations can reduce the risk of infection. Improved surveillance and identification of monkeypox cases is critical to understanding the changing epidemiology of this reemerging and intimidating disease.	Scopus
23	Racial disparity among the clinical outcomes post-myocardial infarction patients. (9)	To estimate clinical outcomes between black and white patients post-MI	220.984 patients	Case report	Case presentation	Odds of all-cause death [OR, 0.71 (95%CI: 0.56–0.91)], *P* = 0.01] and stroke [OR, 0.74 (95%CI: 0.67–0.81)] *P* < 0.001] was significantly lower in the group of white patients compared with black patients	Scopus
24	Monkeypox virus: an emerging epidemic. (23)	To determine the genes responsible for the higher virulence and transmissibility of the virus	Monkeypox cases in Africa in 2022	Case report	Case presentation	Because this virus is zoonotic, there remains great concern regarding the genetic changes of the virus and the risk of its spread to humans.	Scopus
25	Monkeypox-induced myocarditis. (14)	To systematically evaluate the symptoms, imaging findings, management, and outcomes in patients with myocarditis due to monkeypox	9 patients with Myocarditis after monkeypox infection	Case report	Case presentation	That all cases reported occurred in male patients with the most common symptom is chest pain. The overall prognosis is good, with no death reported.	Scopus

Rodriguez-Nava et al. identified two patients with monkeypox who presented with symptoms including dyspnea and chest pain. These patients also exhibited an elevated cardiac biomarker, whereas an echocardiogram revealed no abnormalities on the walls of the ventricles. Additionally, a case report detailed three instances of monkeypox infection and its association with the persistence of cardiovascular inflammation symptoms (23). An additional case report documented an adult male who had a confirmed case of monkeypox and presented with acute myocarditis subsequent to a skin eruption and cardiac magnetic resonance imaging (MRI) diagnosis. Seven confirmed cases of monkeypox were associated with various cardiac complications, including pericardial effusion, pericarditis, myopericarditis, and acute myocarditis, according to a systematic review by Sayad et al. Also reported by Thornhill et al. were two cases of self-limiting myocarditis in patients with monkeypox who recovered without significant complications within seven days (24).

People with behaviour and occupation risk factors for monkeypox infection are considered to get post-prophylaxis therapy, as below. Behaviour risk factors can be men who had sex with other men, gay, transgender, or non-binary people with a recent diagnosis of one or more TDs. Other risk factors include having sex in brothels or at other public events in the last 6 months. Sexual couples from people with risk factors increased the risk of monkeypox infection. Exposure to orthopoxviruses in the workplace also increases the risk of infection in research laboratory personnel and specialised clinical laboratory personnel (25).

Our study discovered that most of the cases were found in male patients and mostly had chest pain. The prognosis was overall good, without any report of death. Infected patients who complain of chest pain must not be ignored, and an appropriate examination should be considered. In the future, further research is needed to predict the occurrence of myocarditis and the following outcomes in patients infected with monkeypox (26).

Myocarditis is an inflammatory condition characterized by cardiac failure, acute arrhythmia, and dilated cardiomyopathy. Whether the myocarditis is self-limiting (resolves within a few weeks), chronic cardiomyopathy (requires long-term therapy), or fulminant (demands circulatory support measures due to critical hemodynamic instability), the prognosis may differ (27). Myocarditis can be induced by a variety of viral pathogens, including enterovirus, adenovirus, or parvovirus B19. The absence of direct viral infection in myocardial cells during histopathology analysis of a sample from a viral vaccine recipient with myocarditis led a number of researchers to hypothesize that the condition was the result of an autoimmune reaction (28).

The primary pathogenesis of viral myocarditis is lymphocyte-associated myonecrosis. Myocarditis can progress to fulminant form or resolve spontaneously within 10–14 days following infection. In the chronic phase of non-infectious viral myocarditis, myocardial fibrosis, heart failure, and dilated cardiomyopathy are hallmark symptoms (29). Due to the rarity of myocarditis cases associated with monkeypox infection, the pathogenesis of myocarditis caused by monkeypox remains unknown. The majority of myocarditis symptoms are mild and self-resolving. Infrequently do severe complications such as dilated cardiomyopathy occur. The infection of myocardium cells by monkeypox is a proposed mechanism for the development of myocarditis in patients with monkeypox. This virus is capable of infecting numerous host cells, including cardiac myocytes, resulting in cell death, inflammation, and injury to the cardiac muscles (30).

Patients with monkeypox may develop myocarditis for a reason that is likely to be attributed to an immunological response to the virus. Upon detecting the virus infection, the immune system secretes cytokines and additional inflammatory mediators, which have the potential to induce myocarditis (26). Severe cases may elicit an uncontrollable immune response, which may initiate cytokine storms that result in extensive tissue devastation, such as damage to the cardiac muscle. In conclusion, the immune response is hypothesised to play a role in the development of myocarditis in patients with monkeypox, which results in direct viral infection of cardiac muscle cells. To obtain a comprehensive understanding of the mechanism and progression of myocarditis in patients with monkeypox, additional research is required (18).

### 5.1 Diagnosis and current management of monkeypox

Although clinical features help differentiate poxvirus illnesses from many further causes of vesiculopustular rashes, laboratory diagnosis is essential for precise identification. Laboratories' investigative procedures for MPX contain IgM, IgG, Enzyme-linked immunoassay (ELISA), electron microscopy, Polymerase Chain reaction (PCR), Virus Isolation, Immuno-fluorescent antibody test, and histopathologic examination. A laboratory with a biosafety level-3 should conduct PCR or real-time PCR. MPXV DNA is routinely detected using real-time PCR using conserved regions of the extracellular envelope protein gene (B6R), DNA polymerase gene, E9l. Rpo18, a DNA-dependent RNA polymerase subunit, and the F3l gene. PCR-amplified genes or gene fragments are also examined by restriction-length fragment polymorphism (RFLP) to detect MPXV DNA. MPXVs and other OPVs remain best characterized by whole-genome sequencing using next-generation sequencing (NGS) methods. Detection of IgG and IgM antibodies are accomplished by ELISA. Immunochemistry analysis can be performed using polyclonal or monoclonal antibodies. Similarly, if accessible, electron microscopy can be used as a laboratory for identifying poxvirus contagions. Under electron microscopy, typical poxvirus virions with the characteristic morphology would be predictable to be detected (31).

The septic person must be isolated, keep covered lesions and wounds, and wear a mask till all lesion crusts break off and a new skin layer form. Interaction among wounded skin or mucous membranes with the bodily fluids, respiratory droplets, or scabs of an infected patient is thought to be a “high risk” contact that necessitates post-exposure vaccination as soon as possible (31). Therapy of MPX infection is symptom control. Supportive therapy may include antipyretics for fever, analgesics for pain, or antibiotics for secondary bacterial infections. However, certain patients may require specific treatment. Those with severe disease, immunocompromised patients, pregnant women, and the pediatric age group may require specific treatment (32).

Due to the similarities MPXV shares with smallpox, drugs and vaccines initially intended to treat smallpox have shown signs of efficacy against MPXV. Tecovirimat is an antiviral medication approved by the United States Food and Drug Administration (FDA) and the European Medicines Agency (EMA) for the treatment of human smallpox disease. Tecovirimat is available in oral (200 mg capsule) and intravenous formulations. According to the Centers for Disease Control and Prevention (CDC), it can be used as a treatment for MPXV in the United States. Cidofovir or brincidofovir can also be used; these are the antiviral medications that the FDA approves for treating cytomegalovirus (CMV) and human smallpox disease, respectively. Vaccinia Immune Globulin Intravenous (VIGIV) is an immunoglobulin used to treat complications (32).

The US CDC allows its use as a treatment for monkeypox disease under an expanded access protocol Vaccination with the Ankara vaccine after vaccinia exposure (live, non-replicating smallpox vaccine) is advised in certain situations. After close connection with an MPX case, it is recommended that vaccination take place within four days after first contact with the virus, but it is possible to give the vaccination up to 14 days after that. A vaccination containing a replication fault, the Ankara vaccine is a two-dose vaccine administered four weeks separately and has a better profile than first- and second-generation smallpox vaccination. Ankara injection, contrasting live vaccinia virus training, does not cause skin lesions or provide a danger of extensive or local transmission (31).

### 5.2 Neurological complications in monkeypox

Only a few of monkeypox's neurological complications have been discovered (see Table 2). Headache is a common symptom that can be found in addition to mood disturbances, including depression, anxiety, and neuropathic pain (22, 33). Skin lesions may cause a painful wound that also depends on the lesion's location. Skin and mucosal lesions may cause dysphagia, anal pain, anal fissures, etc. It is unclear whether some of the pain is dermatome-related, similar to varicella zoster. However, the pain may be severe. Conjunctivitis was found in 20% of patients in the Congo. This may cause a decrease in visual acuity and may be a potential site for the virus to spread into the CNS. Monkeypox rarely causes encephalitis (33).

**Table 2:** Neurological complications from smallpox and monkeypox (37).

Symptoms	Smallpox	Vaccinia vaccine	Monkeypox
Headache	+	+	+
Mood disturbances	−	−	+
Febrile convulsion	+	+	−
Viral encephalitis	+	−	+
Acute disseminated encephalomyelitis	+	+	−
Cranial neuropathies	−	+	−
Transverse myelitis	+	+	−
Acute flaccid paralysis	+	+	−
Guillan-Barre syndrome	+	+	−
Post viral cerebellar signs	+	−	−
Neuropathic pain	−	−	+

An unvaccinated three-year-old girl during the Monkeypox Plague in Zaire from 1980 to 1985 suffered from encephalitis, became coma, and passed away 2 days after hospitalisation (34). During the short plague in West to Central America in 2003, monkeypox was transmitted by pet dogs. A six-year-old girl had several prodromal symptoms such as headache, fever, and malaise, followed by a rash two days later. Seven days from the initial symptoms, the patient had decreased responsiveness, rigidity, mydriatic pupils, pupil edema, and positive Babinski on both sides. Head MRI showed diffuse edema in the cortex, thalamus, and brain stem, as well as an increased contrast in the right parietal and left thalamus. The CSF analysis showed mild pleocytosis (21 cells/mm^3^) with neutrophil dominance (60%), normal protein, and glucose. DNA MPXV (Monkeypox) with a negative PCR test was also found on the CSF. The patient was given supportive care. After being hospitalized for two weeks, the patient was discharged from the hospital. During 1 month of follow-up, the patient had no neurological deficit. The first case of encephalitis was related to clade 1, while the other was related to clade 2. Three cases of encephalitis with seizures were found out of 40 cases in Nigeria, including two patients (a twenty-eight-day-old neonate and a forty-three-year-old man with HIV/AIDS) who later passed away (33, 35).

During this epidemic, three cases of encephalitis had been reported in two Spanish male patients and one Indian male patient, but all of them passed away later. In two Spanish male patients, DNA MPXV was detected by PCR test from CSF analysis, and immunoglobulin M anti-orthodox (IgM) was detected by ELISA (enzyme-linked immunosorbent assay) (35). Until now, the Monkeypox Virus (MPXV) has had two genetically different clades: the Congo Basin clade (CB) and the West Africa clade (WA), with different clinical expressions and geographical locations. The WA clade isn't as severe as the CB clade, with a death rate of 0% to 6%. On the other hand, the CB clade has an 11% death rate, and even higher in children (approximately 17%). Nowadays, the third clade is approved as a part of the WA clade. So that the clade MPXV naming convention did not cause stigma, the clade was then renamed into Clade 1 (CB clade), Clade 2, and Clade 3 (WA clade) (33).

## 6 Conclusion

The emergence of MPOX as a significant public health concern underscores the criticality of investing in infectious disease research and response capabilities and maintaining a high level of awareness. In anticipation of future epidemics of novel viruses and other diseases, it is critical to be ready to address these threats in a timely and efficient manner so as to minimize their negative effects on human health and well-being. With its peak outbreak in 2022 demonstrating dynamic epidemiology and rapid transmission to a significant number of countries that are not endemic regions for this disease, MPOX has emerged as a global public health concern. Among the uncommon complications observed in MPOX cases during the current outbreak is myocarditis. This indicates the necessity for further investigation, the development of efficacious vaccines and antiviral medications, and the implementation of efficient infection prevention and control protocols. The severity of chest symptoms in patients infected with MPXV should not be underestimated; myocarditis should be detected early through screening. When considering the differential diagnosis of myocarditis, it is critical to include MPXV infection, particularly in the context of MPOX outbreaks and events. It is critical to investigate the pathogenesis and clinical characteristics of MPOX, including the virus's capacity to induce myocarditis.

## Data Availability

The raw data supporting the conclusions of this article will be made available by the authors, without undue reservation.
